# A U-Net Deep Learning Framework for High Performance Vessel Segmentation in Patients With Cerebrovascular Disease

**DOI:** 10.3389/fnins.2019.00097

**Published:** 2019-02-28

**Authors:** Michelle Livne, Jana Rieger, Orhun Utku Aydin, Abdel Aziz Taha, Ela Marie Akay, Tabea Kossen, Jan Sobesky, John D. Kelleher, Kristian Hildebrand, Dietmar Frey, Vince I. Madai

**Affiliations:** ^1^Predictive Modelling in Medicine Research Group, Department of Neurosurgery, Charité - Universitätsmedizin Berlin, Berlin, Germany; ^2^Centre for Stroke Research Berlin, Charité - Universitätsmedizin Berlin, Berlin, Germany; ^3^Research Studios Data Science, Research Studios Austria, Salzburg, Austria; ^4^Department of Neurology, Johanna-Etienne Hospital Neuss, Neuss, Germany; ^5^Information, Communication and Entertainment Institute (ICE), Dublin Institute of Technology, Dublin, Ireland; ^6^Department VI Computer Science and Media, Beuth University of Applied Sciences, Berlin, Germany

**Keywords:** cerebrovascular disease, deep learning, medical imaging, segmentation, U-net

## Abstract

Brain vessel status is a promising biomarker for better prevention and treatment in cerebrovascular disease. However, classic rule-based vessel segmentation algorithms need to be hand-crafted and are insufficiently validated. A specialized deep learning method—the U-net—is a promising alternative. Using labeled data from 66 patients with cerebrovascular disease, the U-net framework was optimized and evaluated with three metrics: Dice coefficient, 95% Hausdorff distance (95HD) and average Hausdorff distance (AVD). The model performance was compared with the traditional segmentation method of graph-cuts. Training and reconstruction was performed using 2D patches. A full and a reduced architecture with less parameters were trained. We performed both quantitative and qualitative analyses. The U-net models yielded high performance for both the full and the reduced architecture: A Dice value of ~0.88, a 95HD of ~47 voxels and an AVD of ~0.4 voxels. The visual analysis revealed excellent performance in large vessels and sufficient performance in small vessels. Pathologies like cortical laminar necrosis and a rete mirabile led to limited segmentation performance in few patients. The U-net outperfomed the traditional graph-cuts method (Dice ~0.76, 95HD ~59, AVD ~1.97). Our work highly encourages the development of clinically applicable segmentation tools based on deep learning. Future works should focus on improved segmentation of small vessels and methodologies to deal with specific pathologies.

## Introduction

Stroke is a world disease with extreme impact on patients and healthcare systems. Approximately 15 million people suffer from an ischemic stroke each year worldwide^1^. A third of the patients die, making stroke a leading cause of death. Since stroke is a cerebrovascular disease, more detailed information about arterial vessel status may play a crucial role for both the prevention of stroke and the improvement of stroke therapy. It thus has potential to become a biomarker for new personalized medicine approaches for stroke prevention and treatment (Hinman et al., 2017). Considering that vessel imaging is a routine procedure in the clinical setting, vessel information could be easily integrated in the clinical workflow, if segmentations are available and processed.

Currently, however, vessel imaging is only visually—qualitatively—assessed in the clinical routine. Technical challenges of extracting brain arteries and quantifying their parameters have prevented this information from being applied in the clinical setting. If done at all, segmentations of brain vessels are to date still done predominantly manually or semi-manually and are not quantified. Additionally, (semi-) manual vessel segmentation is very time-consuming and has proven to be fairly inaccurate owing to high interrater-variability making it unfeasible for the clinical setting (Phellan et al., 2017). Consequently, research has focused on developing faster and more accurate automatic vessel segmentation methods. Many different rule-based methods exploiting various features of vessel images, such as vessel intensity distributions, geometric models, and vessel extraction schemes have been proposed for this purpose in the previous decades (Lesage et al., 2009; Zhao et al., 2017). These methods, however, are predominantly manually engineered in nature utilizing hand-crafted features and are—additionally—insufficiently validated (Lesage et al., 2009; Phellan et al., 2017). In fact, due to lack of validation and the necessary performance none of the suggested methods has found any broad use in the clinical setting or in research so far. Thus, crucial information about arterial vessel status and subsequent personalized treatment recommendation are not available. The doctor on site lacks a tool to assess this information for the potential benefit of cerebrovascular disease patients.

Deep neural network architectures are a natural choice to overcome this technological roadblock (Zhao et al., 2017). They have shown tremendous success in the last 5 years for image classification and segmentation tasks in various fields (LeCun et al., 2015; Chen et al., 2016; Badrinarayanan et al., 2017; Krizhevsky et al., 2017), and particularly in neuroimaging (Zaharchuk et al., 2018). In the peer reviewed literature for arterial brain vessel segmentation, Phellan et al. (2017) explored a relatively shallow neural net in magnetic resonance images of 5 patients (Phellan et al., 2017). While showing promising preliminary results, the small sample size and shallow net led to limited performance. Here, one of the most promising deep learning frameworks for segmentation tasks is the U-net (Ronneberger et al., 2015). It is a specialized convolutional neural net (CNN) with an encoding down-sampling path and an up-sampling decoding path similar to an autoencoder architecture. It was specifically designed for segmentation tasks and has shown high performance for the segmentation of biomedical images (Fabijanska, 2018; Huang et al., 2018; Norman et al., 2018).

In this context our central contribution is a modified U-net architecture for fully automated arterial brain vessel segmentation evaluated on a dataset of 66 magnetic resonance (MR) images of patients with cerebrovascular disease. We performed a thorough qualitative and quantitative assessment to assess performance with a special focus on performance for pathological cases. Lastly, we compared our results to a traditional standard method of the graph cut approach (Chen and Pan, 2018).

## Methods

### Patients

We retrospectively used data from patients from the PEGASUS study that enrolled patients with steno-occlusive cerebrovascular disease [at least 70% stenosis and/or occlusion of one middle cerebral artery (MCA) or internal carotid artery (ICA)]. The study details have been published previously (Mutke et al., 2014; Martin et al., 2015). As additional test-sets to assess generalization we included patients with cerebrovascular disease from the 7UP study. Both the 7UP study as well as the PEGASUS study were carried out in accordance with the recommendations of the authorized institutional ethical review board of the Charité-Universitätsmedizin Berlin with written informed consent from all subjects. All subjects gave written informed consent in accordance with the Declaration of Helsinki. The protocol was approved by the authorized institutional ethical review board of the Charité-Universitätsmedizin Berlin.

Of 82 patients in total, 4 did not have vessel imaging and 6 patients were not yet processed at the time of the study. Of the 72 patients remaining, 6 were excluded due to low quality vessel images owing to patient motion leading to 66 patient scans available for our study. The test-sets from the 7UP study comprised 10 patients each with cerebrovascular disease (stroke in the past) with Time-of-Flight (TOF)-angiography from a different scanner and different parameters, and a different angiography modality, i.e. MPRAGE-angiography (Dengler et al., 2016).

### Data Accessibility

At the current time-point the imaging data cannot be made publicly accessible due to data protection, but the authors will make efforts in the future, thus this status might change. Researchers interested in the code and/or model can contact the authors and the data will be made available (either through direct communication or through reference to a public repository).

### Imaging

For the PEGASUS patients, scans were performed on a clinical 3T whole-body system (Magnetom Trio, Siemens Healthcare, Erlangen, Germany; in continuation referred to as Siemens Healthcare) using a 12-channel receive radiofrequency (RF) coil (Siemens Healthcare) tailored for head imaging.

Time-of-Flight (TOF) vessel imaging was performed with the following parameters: voxel size = (0.5 × 0.5 × 0.7) mm^3^; matrix size: 312 × 384 × 127; TR/TE = 22 ms/3.86 ms; time of acquisition: 3:50 min, flip angle = 18 degrees.

For the additional 7UP test-sets, scans were performed on a clinical 3T whole-body system (Magnetom Verio, Siemens Healthcare) and a 12 channel RF receive coil (Siemens Healthcare) for TOF-imaging and a 7T whole-body system (Magnetom 7.0 T, Siemens Healthcare) with a 90 cm bore magnet (Magnex Scientific, Oxfordshire, United Kingdom), an avanto gradient system (Siemens Healthcare) and a 1/24 channel transmit/receive coil (NovaMedical, Wakefield, MA) was used for MPRAGE-angiography.

The parameters were:

TOF imaging: voxel size = (0.6 × 0.6 × 0.6) mm^3^; matrix size: 384 × 384 × 160; TR/TE = 24 ms/3.60 ms; time of acquisition: 5:54 min, flip angle = 18 degrees.

MPRAGE imaging: voxel size = (0.7 × 0.7 × 0.7) mm^3^; matrix size: 384 × 384 × 240; TR/TE = 2,750 ms/1.81 ms; time of acquisition: 5:40 min, flip angle = 25 degrees.

### Data Postprocessing

The raw PEGASUS study TOF images were denoised using the oracle-based 3D discrete cosine transform filter (ODCT3D) implemented in matlab (Manjón et al., 2012). Non-uniformity correction (NUC) was performed with the *mri_nu_correct.mni* tool integrated in freesurfer (website: Freesurfer mri_nu_correct.mni)^2^. Corresponding whole-brain masks were automatically generated using the *Brain Extraction Tool (BET)* of FSL (website: BET/UserGuide-FslWiki)^3^. Both NUC and FSL-BET post-processing were performed with the *Nipype* wrapper implemented in Python^4^. The post-processing parameters were as follows: ODCT Filter: Patch size 3 × 3 × 3 voxels, Search volume size: 7 × 7 × 7 voxels, Rician noise model; Freesurfer mnibias correction: iterations = 6, protocol_iterations = 1,000, distance = 50; FSL BET: frac = 0.05.

The additional 7UP TOF and MPRAGE imaging test-set image pipeline differed in these points: non-local means denoising implemented in Nipype (patch radius = 1, block radius = 5, and Rician noise model) and MPRAGE-BET parameters (frac 0.15).

### Data Labeling

For PEGASUS TOF data, ground-truth labels of the brain vessels were generated semi-manually using a standardized pipeline. Pre-labeling of the vessels was performed by a thresholded region-growing algorithm using the *regiongrowingmacro* module implemented in MeVisLab (website: MeVisLab)^5^. To tackle inter-rater variability in label generation, these pre-labeled data were thoroughly manually corrected by either OUA and EA (both junior raters) and then cross-checked by the other rater. These labels were then checked subsequently both by VIM (9 years experience in stroke imaging) and DF (11 years experience in stroke imaging). Thus, each ground-truth was eventually checked by 4 independent raters, two of them senior raters. The total labeling time with this framework amounted to 60–80 min per patient.

Additional test-set label data (TOF and MPRAGE imaging) was generated using the U-net model in a first step instead of the *regiongrowingmacro* framework, followed by the above described thorough manual correction steps. Images were reviewed in the final step by VIM.

### Data Splitting

**Figure 1:**
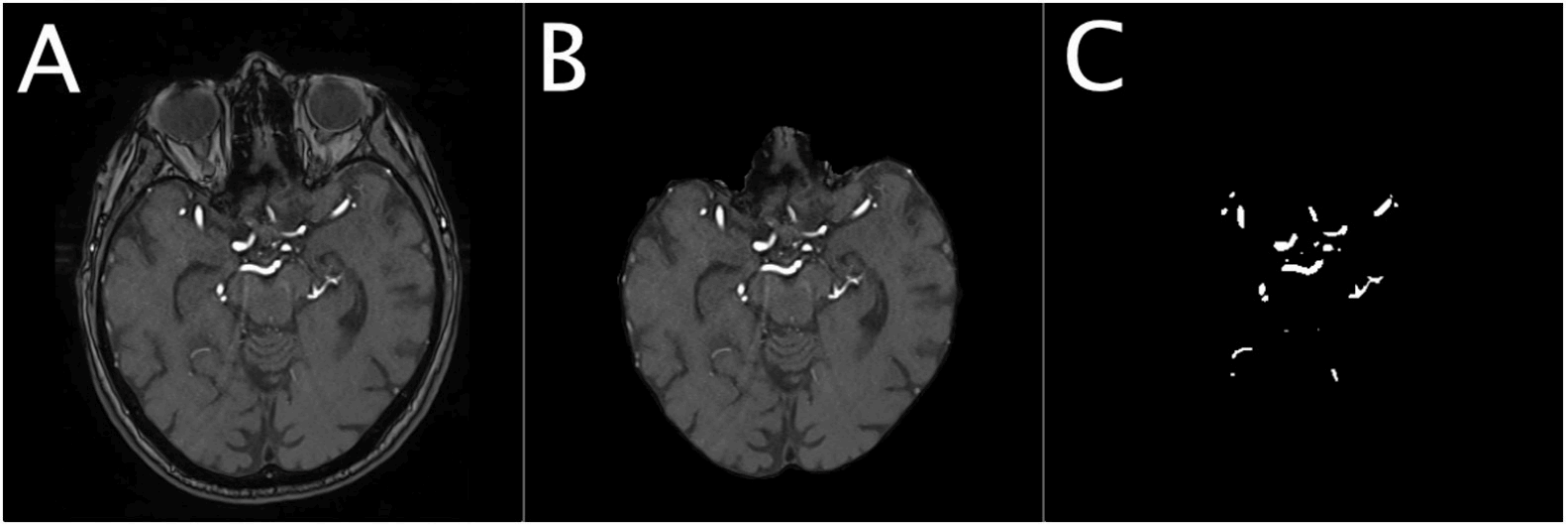
Illustration of the dataset for a representative patient. The Figure shows an illustration of the denoised TOF image of a representative patient **(A)**, the corresponding masked brain image **(B)** and the corresponding ground-truth image of the brain vessels **(C)**.

For U-net training, both PEGASUS TOF images and ground-truth labels were skull-stripped using the whole-brain masks. The data was split into training, validation, and test-sets with 41, 11, and 14 patients-scans, respectively. For illustration of the extracted data see Figure 1.

### Random Patch Extraction

In order to successfully train our deep neural network we needed to consider two challenges. First, the brain slices, with 312 × 384 voxels, are very large and cannot processed at once due to the limited GPU memory. Second, the distribution of vessels within a slice is largely skewed as only 0.9% of brain voxels are vessels. To solve these problems we extracted 1,000 quadratic patches per patient: 500 random patches with a vessel in the center and 500 random patches without a vessel in the center. The model was trained using 4 different patch sizes (16 × 16, 32 × 32, 64 × 64, 96 × 96 voxels) and was later tested for best results against the validation set as part of the optimization process. Due to computational limitation the maximal patch size was set to 96 × 96 voxels. Testing the effect of different patch sizes on the model performance would reveal important information about the relevant spatial scope for a reliable vessel detection. The data was normalized patch-wise using zero-mean and unit-variance normalization.

### Network Architecture and Training

#### Network Architecture

**Figure 2:**
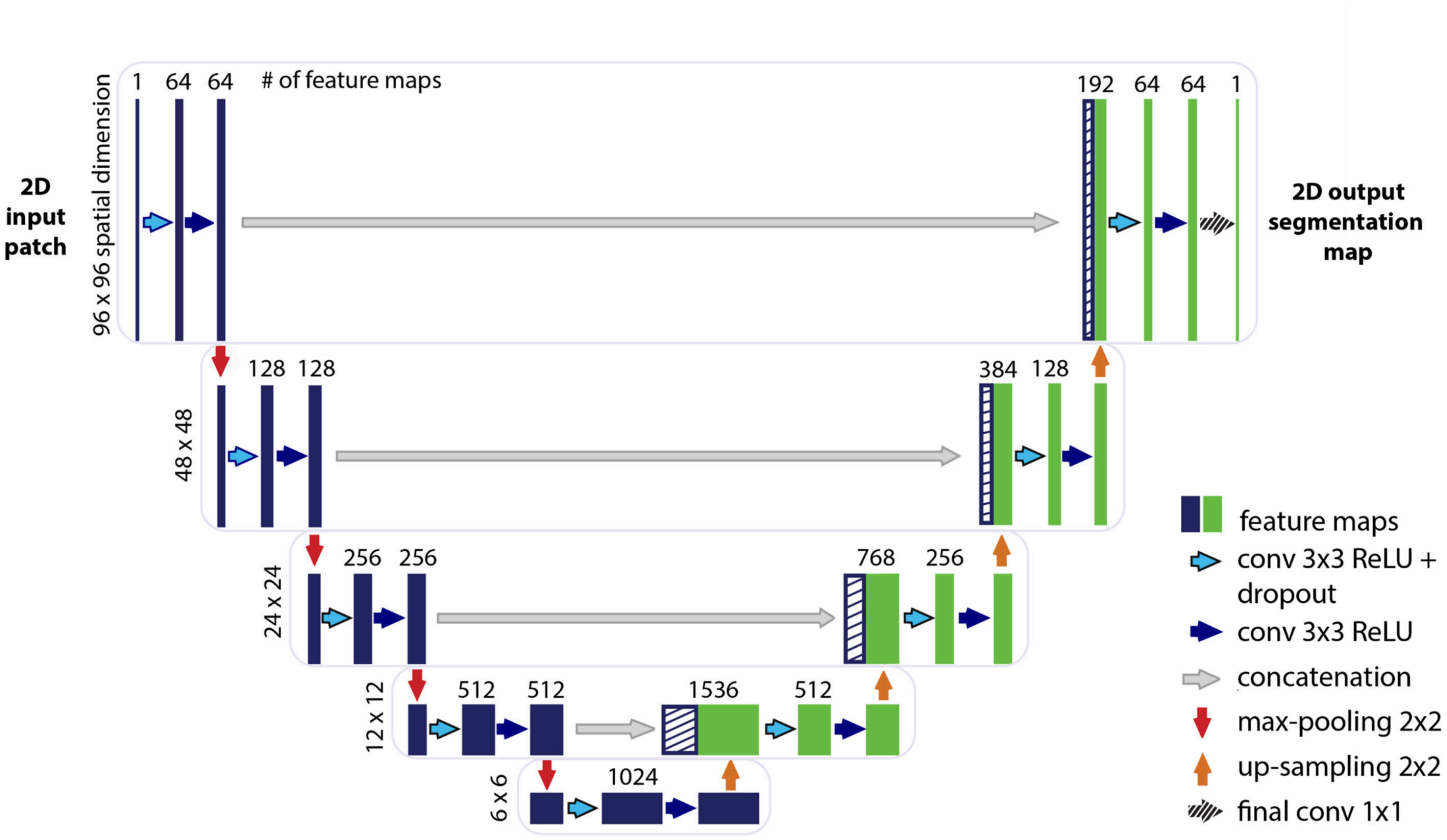
Illustration of the U-net architecture. The figure illustrates the U-net architecture with the largest patch-size of 96 × 96 voxels. The displayed U-net is an encoder-decoder network with a contracting path (encoding part, left side) that reduces the height and width of the input images and an expansive path (decoding part, right side) that recovers the original dimensions of the input images. Each box corresponds to a multi-channel feature map. The dashed boxes stand for the concatenated copied feature maps from the contractive path. The arrows stand for the different operations as listed in the right legend. The number of channels is denoted on top of the box and the image dimensionality (x-y-size) is denoted on the left edge. The half U-net is constructed likewise, with the only difference given by the halved number of channels throughout the network.

The U-net CNN model architecture was adapted from the framework presented by Ronneberger et al. (2015). The U-net model architecture is shown in Figure 2. The network is based on a convolutional neural network (CNN) and consists of an encoding and a decoding part. The contracting path, i.e., encoding part (left side) repeatedly applies two (padded) 3 × 3 convolutional layers with stride 1, each followed by a rectified linear unit (ReLU) and a 2 × 2 max-pooling operation with stride 2 on 4 levels. A dropout layer is applied following the first convolutional layer. At each down-sampling step the dimensions of the input image is reduced by half and the number of feature channels is doubled. The bottom level includes two 3 × 3 convolutional layers without pooling layer. The expansive path, i.e., decoding part (right side) recovers the original dimensions of the input images by up-sampling the feature map, a concatenation with the corresponding feature channels from the contractive path and two 3 × 3 convolutional layers, the first followed by ReLU and a dropout-layer and the second followed by ReLU. The final layer is a 1 × 1 convolution for mapping the feature vector to the binary prediction (i.e., vessel vs. non-vessel).

A variation of the U-net model architecture was applied, where the number of channels in each layer was consistently reduced to half. For simplicity, the additional architecture is therefore termed throughout this work as “half U-net.”

The network is fed with 2D image patches and returns the 2D segmentation probability map for each given patch.

#### Model Training

The skull-stripped denoised TOF input images and the corresponding ground-truth segmentation maps were used to train the U-net using the Keras implementation of Adam optimizer (Kingma and Ba, 2014).

In the model, the energy function is computed by a pixel-wise sigmoid over the final feature map combined with the Dice coefficient loss function. The sigmoid is defined as *p*(*x*) = 1/(1 + exp(*a*(*x*))) Where *a*(*x*) denotes the activation in the final feature channel at the voxel position *x* ∈ *Ω* with *Ω* ∈ *Z*^2^ and *p*(*x*) is the approximated probability of a voxel *x* to be a vessel. The Dice coefficient *D* between two binary volumes is officially defined as $D=\frac{2TP}{2TP+FP+FN}$ Where *TP* is the number of true-positive voxels, *FP* is the number of false positive voxels and *FN* is the number of false negative voxels. Using the following derivation:

$$\begin{array}{l} D=\frac{2TP}{2TP+FP+FN}\\ \;=\frac{2\sum_{x\in \Omega }p_{x}g_{x}}{2\sum_{x\in \Omega }p_{x}g_{x}+\sum_{x\in \Omega }\left(p_{x}^{2}-p_{x}g_{x}\right)+\sum_{x\in \Omega }\left(g_{x}^{2}-p_{x}g_{x}\right)} \end{array}$$

The Dice coefficient can be written as:

$$\begin{array}{lll} D & = & \frac{2\sum_{x\in \Omega }p_{x}g_{x}+s}{\sum_{x\in \Omega }p_{x}^{2}+\sum_{x\in \Omega }g_{x}^{2}+s} \end{array}$$

Where *p*_*x*_ ∈ *P*:Ω → {0, 1} is the predicted binary segmentation volume, *g*_*x*_ ∈ *G*:Ω → {0, 1} is the ground-truth binary volume and *s* = 1 is an additive smoothing factor (i.e., Laplace smoothing). The Dice coefficient then penalizes at each position *j* the deviation of *p*_*x*_ from the true label *g*_*x*_ using the differentiated gradient:

$$\begin{array}{lll} \frac{\partial D}{\partial p_{j}} & = & 2\left[\frac{g_{j}\left(\sum_{x\in \Omega }p_{x}^{2}+\sum_{x\in \Omega }g_{x}^{2}\right)-2p_{j}\left(\sum_{x\in \Omega }p_{x}g_{x}\right)}{\left(\sum_{i\in \Omega }p_{x}^{2}+\sum_{x\in \Omega }g_{x}^{2}+s\right)}\right] \end{array}$$

(Milletari etal., 2016)

The choice of the Dice coefficient as the loss function allows to handle the skewed ground-truth labels without sample weighting.

A constructive initialization of the weights is necessary to ensure gradient convergence, while preventing the situation of “dead neurons,” i.e., parts of the network that do not contribute to the model at all. This is particularly true for the case of deep neural networks with many convolutional layers and many different paths through the network. Here we applied the commonly used heuristic with ReLU activation function, the Glorot uniform initializer where the initial weights are drawn from a uniform distribution within the range [−*L, L*] where *L*$=\sqrt{6/\left(f_{in}+f_{out}\right)}$, *f*_*in*_ is the number of input units in the weight tensor and *f*_*out*_ is the number of output units in the weight tensor (Glorot and Bengio, 2010).

The models were tuned in the validation process to optimize the hyperparameters learning-rate, batch-size, and dropout-rate in addition to the optimization of the patch-size as described above.

#### Data Augmentation

Augmentation methods introduce variance to the training data which allows the network to become invariant to certain transformations. While CNNs and U-net in specific are very good in integration of spatial information which is essential to imaging segmentation tasks, they are not equivariant to transformations such as scale and rotation (Goodfellow et al., 2016). Data augmentation methods like rotations and flips yield the desired invariance and robustness properties of the resulted network. Additionally to flips and rotations, the data augmentation included shears as a derivative of elastic deformations which are recommended as general best practice for convolutional neural networks (Ronneberger et al., 2015). The augmentation was applied on-the-fly on the patch-level using the *ImageDataGenerator* function implemented in Keras.

### Method Comparison

For comparison we used the graph cut implementation in the PyMaxFlow Python library in Version 1.2.11 (Neila, 2018). This method is tailored for binary segmentation problems, where the combination of Markov Random Fields (MRF) with Bayesian maximum a posteriori (MAP) estimation turns the segmentation task into a graph based minimization problem. Then, the graph cut methodology provides a computationally efficient solution to the minimization problem (Chen and Pan, 2018). We tuned the *weights* hyperparameter, representing the uniform capacity of the edges, on the validation set to determine the optimal setting. With this setting the algorithm was applied on the 14 patients of the test-set to produce segmentation images. We applied both patch wise segmentation with a patch size of 96 as well as segmentation whole slice by slice.

### Performance Assessment

#### Quantitative Assessment

The model performance was assessed based on three different measures: The Dice coefficient, 95HD and the AVD. While the Dice coefficient serves as a general common measure for segmentation tasks, the 95HD and AVD metrics allow to capture more accurate estimation of performance with relation to the boundary error of the branched and complex structure of brain vessels. In contrast to the Hausdorff distance which relates to the maximum of the distance metrics, the 95HD and AVD are calculated as the 95% percentile- and the average distance, respectively.

Therefore, 95HD and AVD are stable and not sensitive to outliers which is typically an important quality measure in medical images analysis. While the Dice coefficient ranges from [0,1] unitless values where the larger the value, the better performance it indicates, the units of 95HD and AVD represent real distances with voxels as a unit, hence the smaller the value is, the better the performance is. The measures were calculated using the EvaluateSegmentation tool provided by Taha and Hanbury (2015), Taha (2018). We identified three final models for performance comparison: Since we based our assessment on three different metrics—the Dice coefficient, the 95HD and the AVD—we chose a model that optimized each of the metrics based on the validation set. The performance was assessed as an average of the measures of all fully reconstructed vasculatures of the patients in the test-set as well as on the segmentations resulting from the graph cut approach.

#### Qualitative Assessment

For qualitative assessment the predicted segmentation masks as well as the graph cut results of the 14 patients in the test-set were transformed by an in-house python code where true positives (TP), false positives (FP), and false negatives (FN) were assigned different voxel values (True negatives (TN) remained labeled with 0). The images were then visualized by overlaying these new masks with the original scans using ITK-Snap (website: ITK-SNAP)^6^. By adjusting the opaqueness, it was possible to qualitatively assess which structures were correctly identified and which anatomical structures dominated with errors. For each architecture and each model (2 architectures × 3 models = 6) VIM visually assessed per patient the images based on a predefined scheme. Large vessels were defined as the all parts of the ACA and the M1, A1, and P1 segments of the three large brain arteries. All other parts were considered small vessels. The results of the visual analysis are summarized qualitatively in the results section. The scheme was the following:

Large vessels, overall impression (bad, sufficient, good); Small vessels, overall impression (bad, sufficient, good); Large vessels, errors (FP or FN dominating); Small vessels, errors (FP or FN dominating); Pathology detected (yes/no); Other tissues type segmentation errors (yes/no).

### Generalization Assessment

All 6 models were applied on the additional 10 7UP patients with different TOF parameters as well as 10 patients with a different angiography modality (7T MPRAGE angiography). Segmentation quality was compared vs. the semimanual gold-standard labels as described above quantitatively with the EvaluateSegmentation framework using as metrics Dice, 95HD and AVD.

### Hardware

All trainings ran on a GPU workstation with 16 GB RAM, Intel(R) Xeon(R) CPU E3-1231 v3 @ 3.40GHz and a NVidia TITAN X (Pascal) GPU with 12 GB VRAM.

## Results

**Figure 3:**
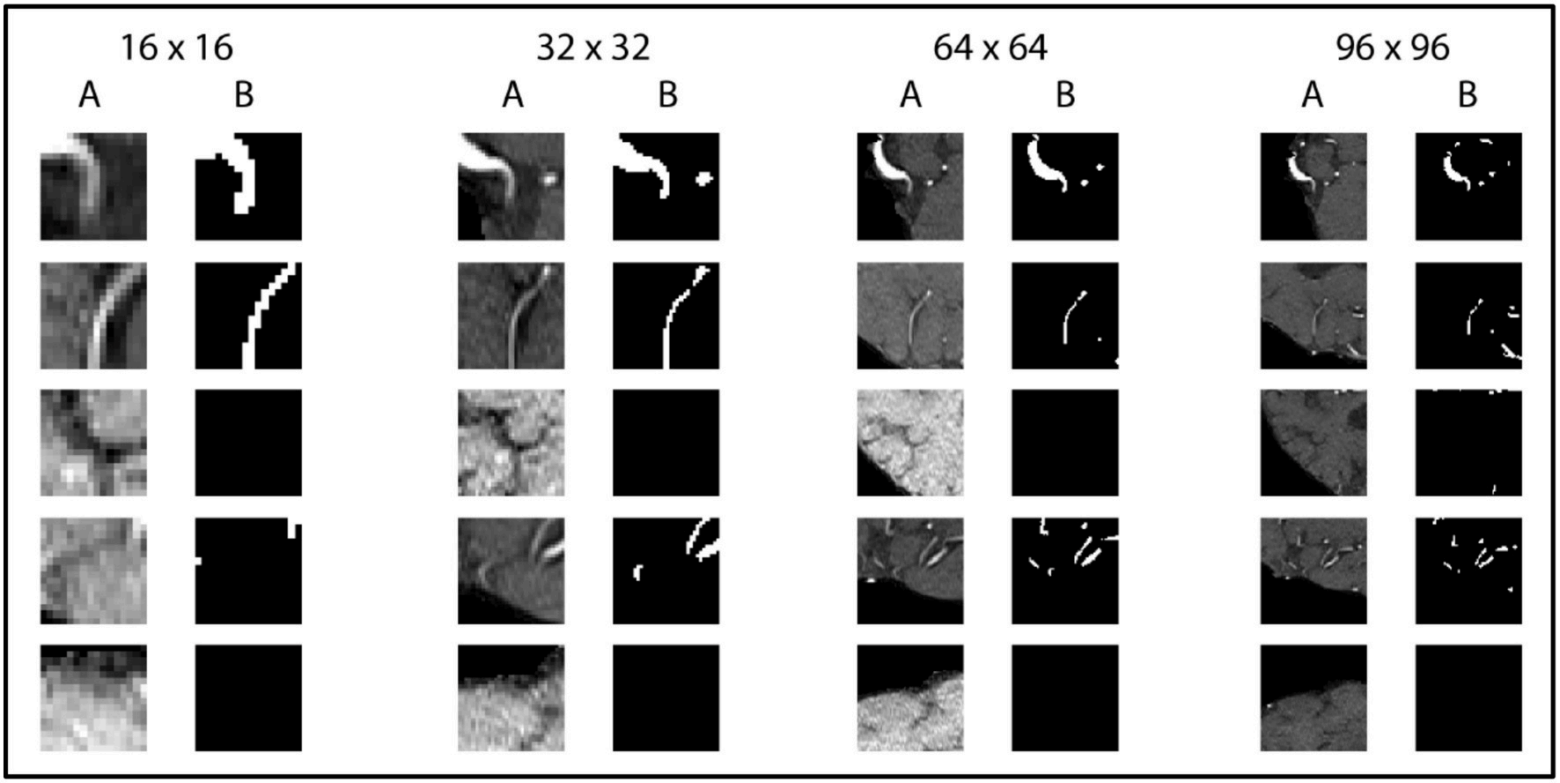
Exemplary patches used for training. Five pairs of random patches with increasing patch size from left to right are shown. “A” columns indicate the MRA-TOF-scans, whereas “B” columns indicate the ground truth label.

The U-net model was trained on 81,000 extracted and augmented patches of 41 patients, validated using 11 full patient volumes and assessed for performance using the test-set of 14 full patient volumes. The U-net architecture resulted in 31,377,793 parameters, while the half U-net resulted in 7,846,081 parameters. For the U-net and half U-net, respectively, model training ran for ~100 and 50 min, while segmentation of a previously unseen volume took about 20 and 10 s. The optimal patch-size was identified as 96 × 96 voxels for both suggested architectures. The model parametrization can be found in Table 1. Exemplary patches used for training can be seen in Figure 3.

**Table 1:** Model parametrization.

**Optimized performance measure/ Hyperparameter**	**Dice coefficient**	**95% Hausdorff distance**	**Averaged Hausdorff distance**
**U-NET MODEL PARAMETRIZATION**			
Learning rate	1e-4	1e-4	1e-5
Batch size	16	64	8
Dropout rate	0	0	0
**HALF U-NET MODEL PARAMETRIZATION**			
Learning rate	1e-4	1e-4	1e-4
Batch size	64	32	32
Dropout rate	0.1	0.2	0.1
**GRAPH CUT PARAMETRIZATION**			
**Hyperparameter**	**Value**		
Weights	10		

**Figure 4:**
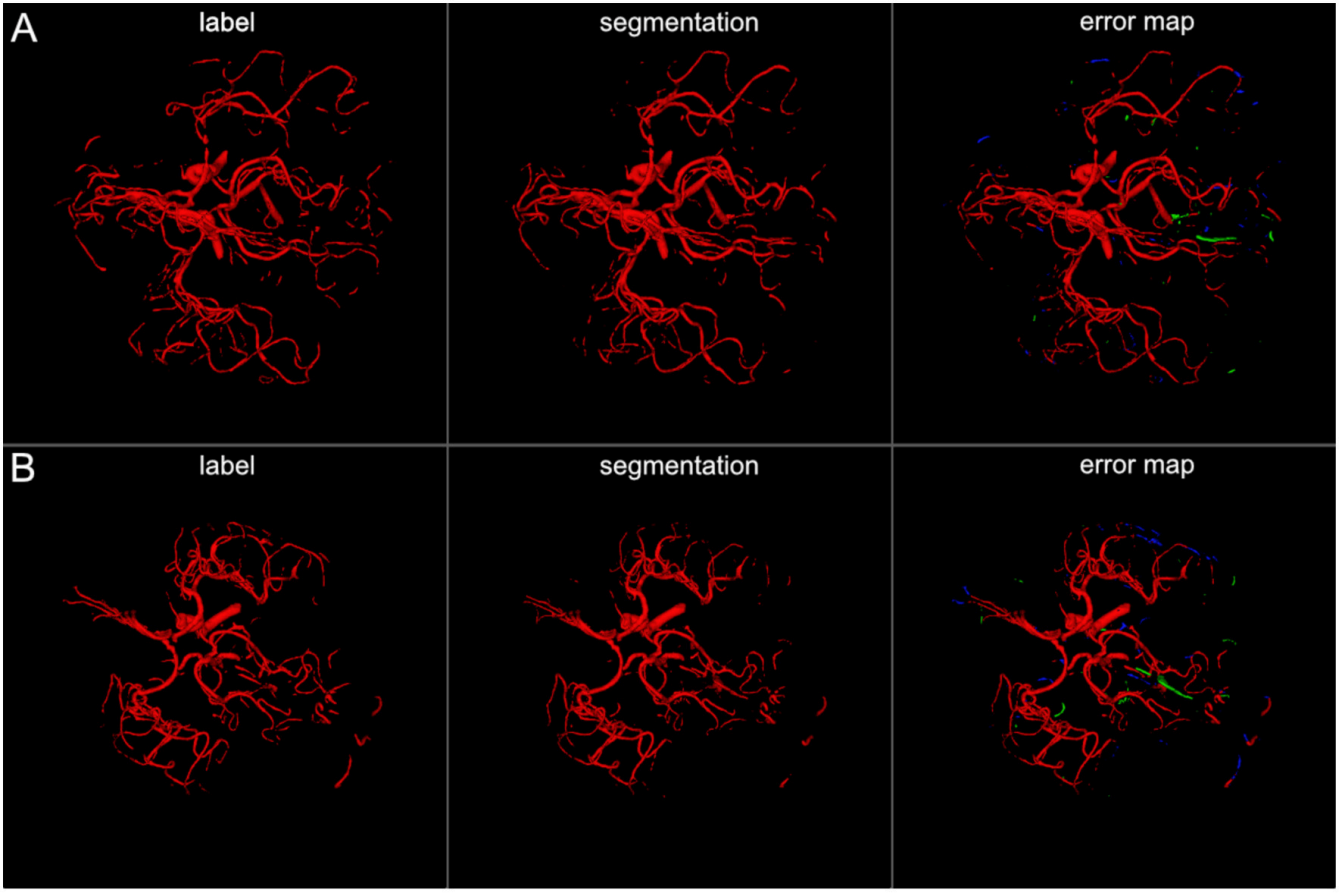
3D projections of segmentation results. The figure illustrates exemplary segmentation results as 3D projections for two representative patients **(A,B)**. Labels are shown in the first column and exemplary segmentation results are shown in the second column. The third column shows the error map, where red voxels indicate true positives, green voxels false positives, and blue voxels false negatives. Overall a high performance segmentation could be achieved. In the error maps it can be seen that false positives mainly presented as central venous structures and parts of meningeal arteries. (3D view is meant for an overall overview. Due to 3D interpolation, very small structures may appear differently in the images. This does not translate to real voxel-to-voxel differences. For direct voxel-wise comparison please use the 2D-images in Figures 5–8).

The U-net model yielded high performance in terms of the three measures that were comparable for both the full and the half architecture: The models optimized for the Dice coefficient had a Dice value of 0.89. The 95HD value was 47 voxels and the AVD models yielded results around 0.35 voxels. The detailed performance assessment of the finalized models is presented in Table 2. A representative overview of the visual analysis can be found in Figure 4.

**Table 2:** Summary of test-set performance measures.

**Model architecture/Performance measure**	**U-net**	**Half U-net**	**Graph cut (patch/whole slice)**
Dice coefficient	0.891	0.892	0.758/0.758
95% Hausdorff distance (voxels)	47.277	47.099	58.79/59.24
Averaged Hausdorff distance (voxels)	0.342	0.385	1.965/1.974

*Detailed performance measures of the two Dice-optimized U-net model architectures and the baseline graph-cuts segmentation model as the averaged value over the 14 test patients. For the detailed models parametrization, see Table 1*.

**Figure 5:**
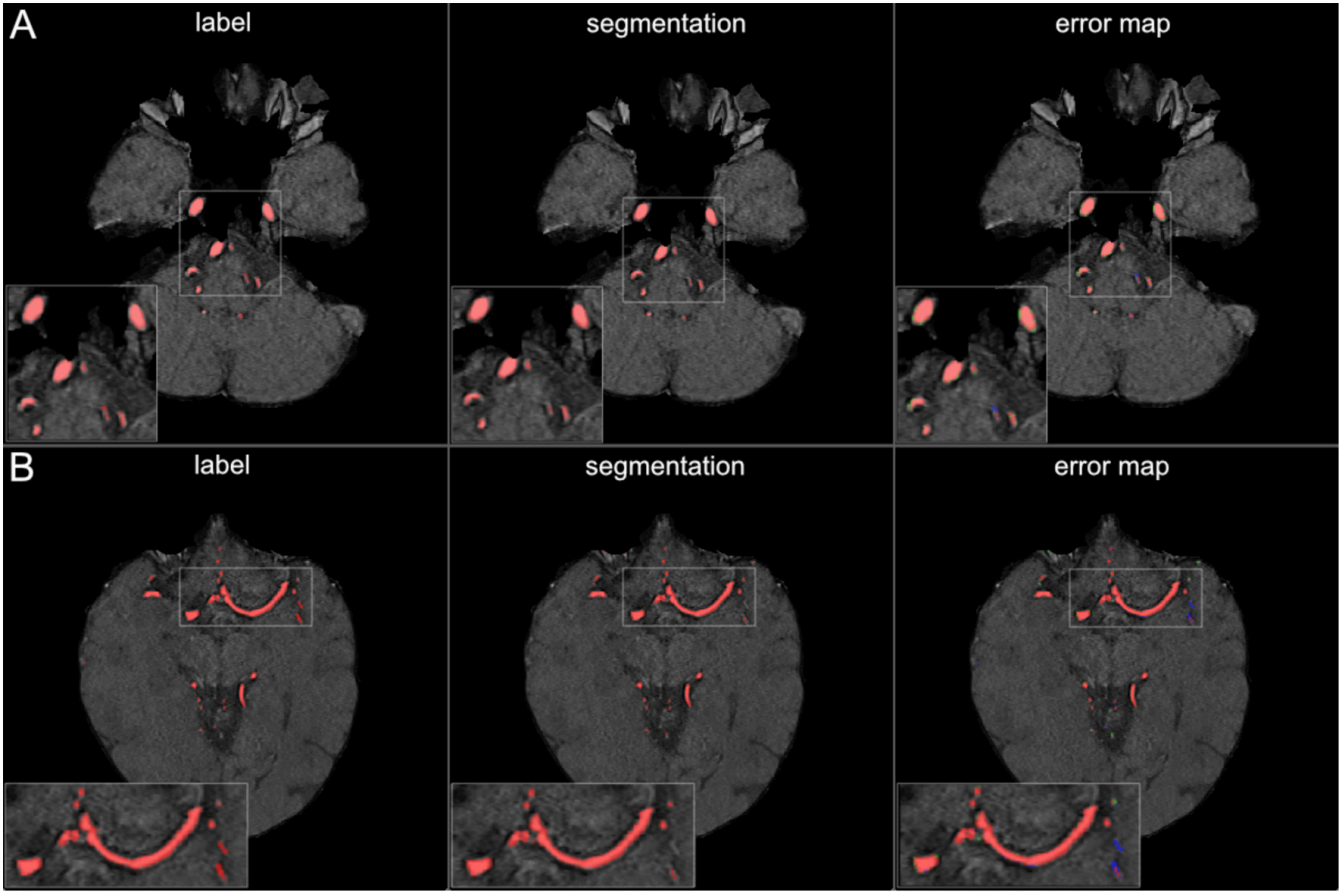
Segmentation results for large vessels. The figure illustrates exemplary segmentation results for large vessels for two representative patients **(A,B)**. Labels are shown in the first column and exemplary segmentation results are shown in the second column. The third column shows the error map, where red voxels indicate true positives, green voxels false positives, and blue voxels false negatives. Only few false positive voxels can be seen in the border zones of the vessels.

**Figure 6:**
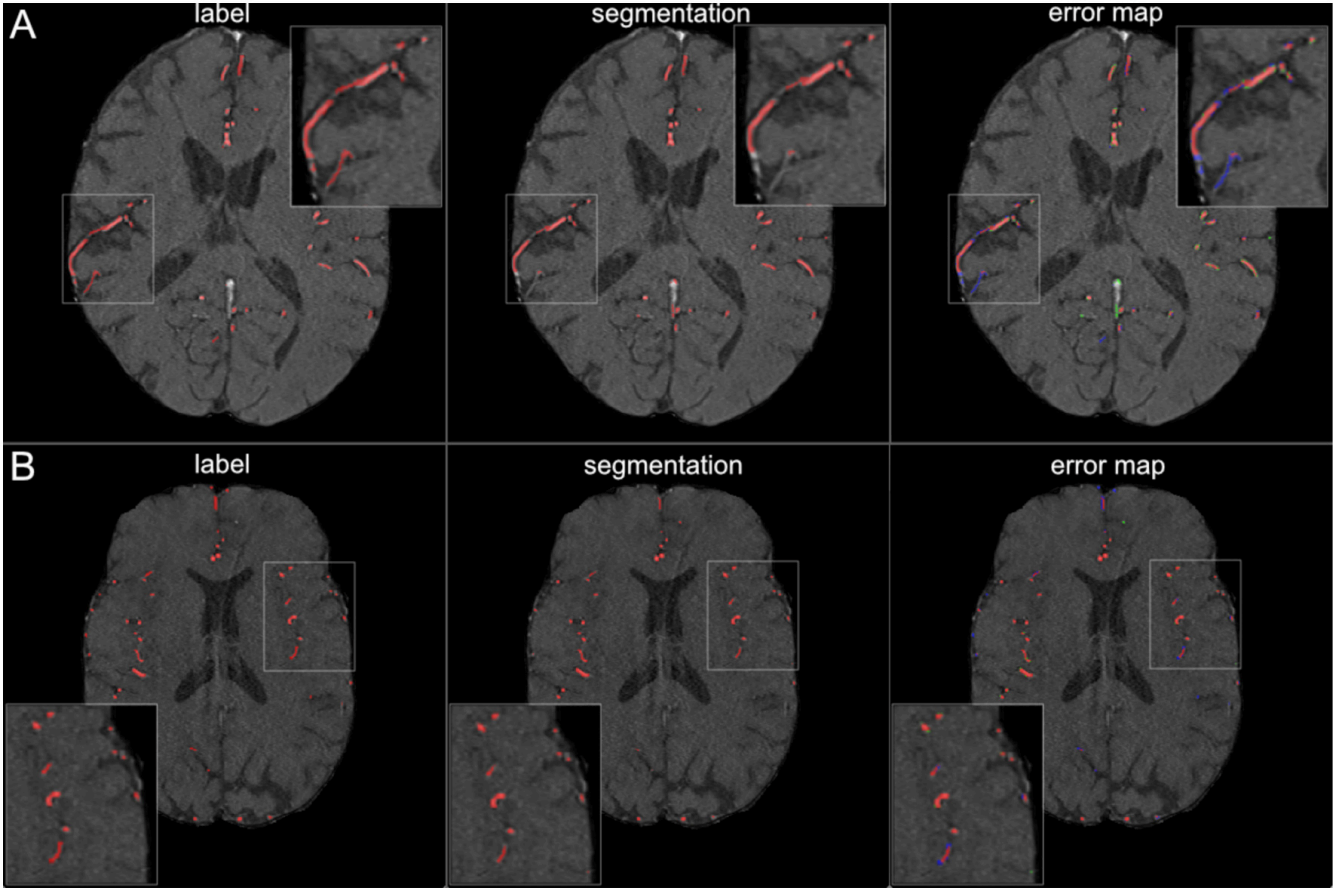
Segmentation results for small vessels. The figure illustrates exemplary segmentation results for small vessels for two representative patients **(A,B)**. Labels are shown in the first column and exemplary segmentation results are shown in the second column. The third column shows the error map, where red voxels indicate true positives, green voxels false positives and blue voxels false negatives. In 7 patients (50%) also small vessels were segmented well, with only few false negatives **(B)**. In the other patients, the small vessels were segmented only sufficiently, with both false positives and false negatives **(A)**.

**Figure 7:**
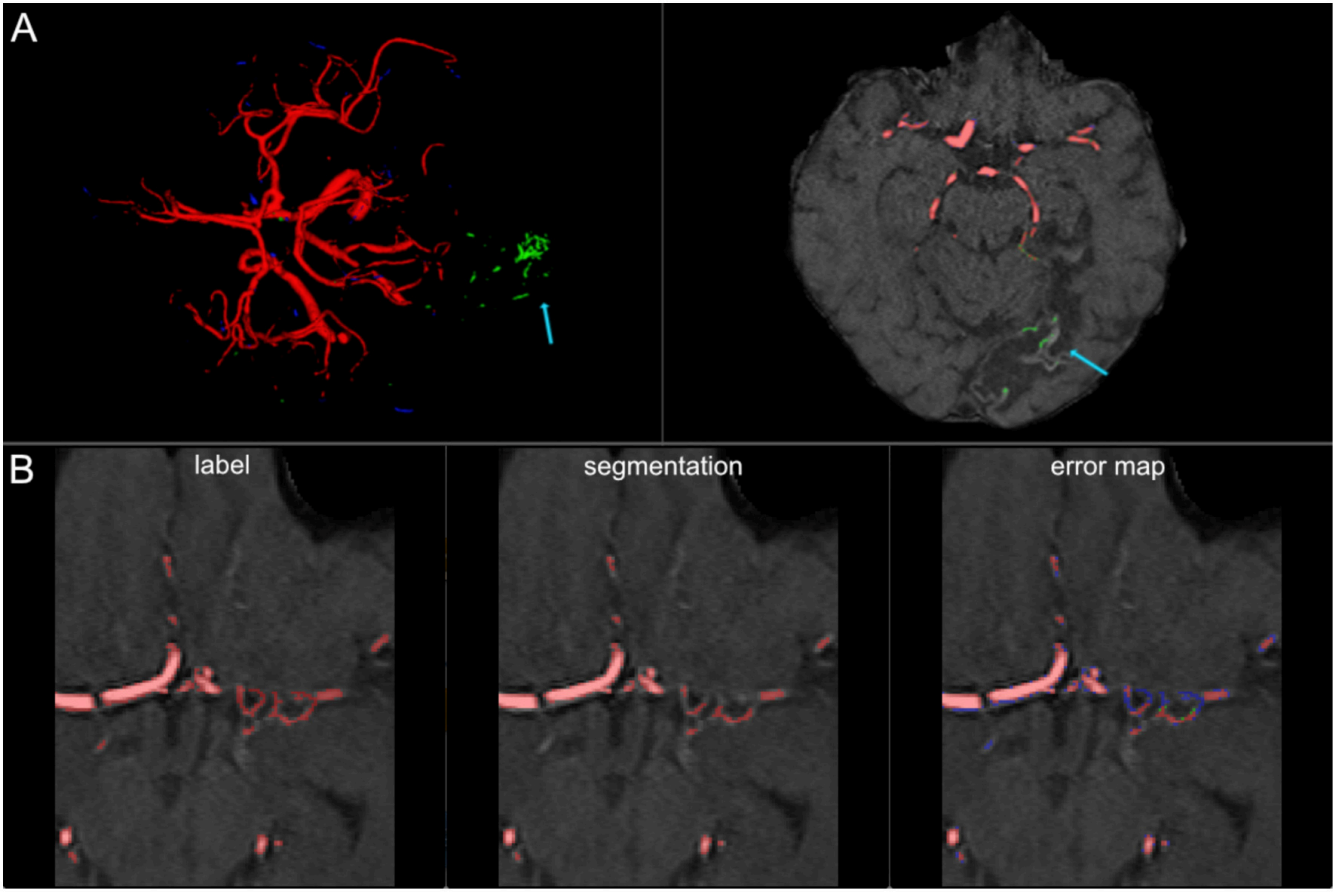
False labeling of specific cerebrovascular pathologies. **(A)** The error map of a 3D projection on the left shows falsely labeled structures in the posterior part of the brain (arrow). On a transversal slice (on the right) false labeling of parts of cortical laminar necrosis can be identified as the cause (arrow). **(B)** The rete mirabile network of small vessels was only partially depicted (false negative labeling in blue in the error map). A rete mirabile is a relatively rare occurrence, only 3 patients of 66 in our study presented with one (2 in the training set and one in the test-set).

The visual analysis of the three full models showed that consistently in all 14 patients the large vessels were segmented excellently. Only very few false positive voxels in the border zones of the vessels were present (see Figure 5). Small vessels were segmented well in half of the patients, in the other patients small vessels were segmented less well (see Figure 6). In 12 patients, we found false positive labeling of small parts of meningeal arteries present in the image or of venous structures (sinus and central veins) (see Figures 4, 6). In one patient, tissue in an old infarct presented as cortical laminar necrosis with hyperintense elongated tissue against the dark cerebrospinal fluid. These parts were partially labeled as vessels (see Figure 7A). In another patient, a rete mirabile, a vessel network of small arteries developing due to occlusion, was present. The rete mirabile was only partially segmented (see Figure 7B).

A comparison of the three models showed comparable performance and consistent artifacts as described above. There was a tendency, however, for 95HD and AVG models to have less false positively labeled meningeal and venous structures than the Dice-optimized model. The visual comparison of the full and the half architecture showed comparable performance in the large vessels. We saw a tendency for slightly worse performance in smaller vessels in the half architectures. Vessel pathologies (stenosis/occlusion) were depicted in all patients and all models.

**Figure 8:**
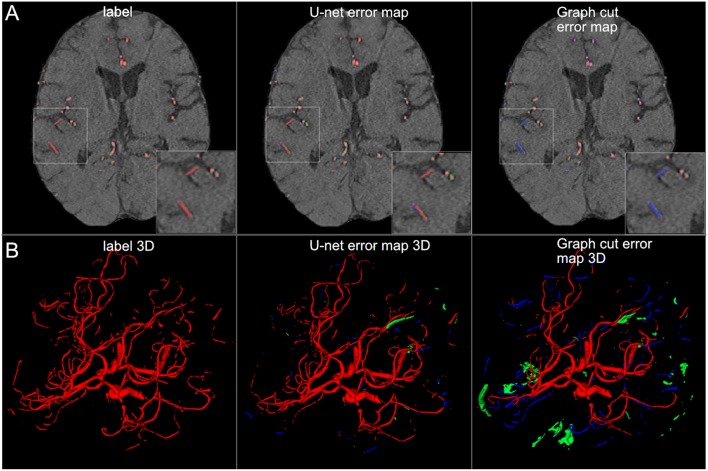
Comparison of U-net and graph cut segmentations. **(A)** 2D comparison and **(B)** 3D comparison of labels, U-net error maps and graph cut error maps. The quantitative results are confirmed by visual inspection. The graph cut segmentation shows more false negatively (blue) and false positively (green) segmented voxels.

Graph cut results showed inferior performance to the U-net models with the following results (patch/whole slice): Dice 0.76/0.76, 95 HD 58.8/59.2, and AVD 1.97/1.97 (Detailed results can be found in Table 2 and a visual example in Figure 8).

Generalization assessment showed a very good performance of the Dice optimized models for the intra-modal comparison with 3T TOF images with a Dice of 0.86 / 0.92, 95 HD of 64.5 / 50.0, and AVD of 1.591 / 0.650 for the full U-net and half U-net, respectively. We found insufficient performance for inter-modal comparison with 7T MPRAGE angiography (Dice around 0.60, 95 HD around 50, and AVD around 3.5). Detailed results can be found in Table 3.

**Table 3:** Summary of generalization assessment results.

	**U-net**	**Half U-net**
**3T TOF ANGIOGRAPHY:**		
Dice coefficient	0.858	0.921
95% Hausdorff distance (voxels)	64.535	50.015
Averaged Hausdorff distance (voxels)	1.591	0.650
**7T MPRAGE ANGIOGRAPHY:**		
Dice coefficient	0.594	0.661
95% Hausdorff distance (voxels)	54.598	48.414
Averaged Hausdorff distance (voxels)	3.467	3.489

*Detailed performance measures of the two tested model architectures as the averaged value over 10 test patients each for 3T TOF and 7T MPRAGE angiography. The results for the two Dice optimized models are shown. The half U-net architecture exhibited better generalization performance*.

## Discussion

We present in the current work a U-net deep learning framework for fully automated brain arterial vessel segmentation from TOF-images of patients with cerebrovascular disease. Our framework demonstrated a very high quantitative performance based on three validation metrics. A lighter architecture—half U-net—achieved comparable quantitative performance. Visual inspection showed excellent performance in large vessels and sufficient to good performance in small vessels as well as comparable performance between the full architecture and the half-net. Special cerebrovascular pathologies presented challenges for the network and need to be addressed in the future.

Applying a modified U-net framework as suggested by Ronneberger et al. (2015), we achieved a very high quantitative performance for the segmentation of arterial brain vessels in patients with cerebrovascular disease. To the best of our knowledge, our work is the first study to show the value of a U-net architecture for fully automated arterial brain vessel segmentation in cerebrovascular disease. Our results are therefore highly encouraging for the further development of automated clinical vessel segmentation tools for cerebrovascular disease. In contrast to the so called “rule-based” non-neural-net attempts of the past, deep learning based networks do not require hand-crafted features or prior feature selection (Lesage et al., 2009; Zhao et al., 2017). The main reason for this is the inherent ability of U-nets to efficiently extract the relevant features in the training process. Confirming the broad consensus that deep learning based approaches constitute the new state-of-the-art in medical segmentation, the U-Net architecture clearly outperformed the traditional graph-cut based segmentation method.

Next to a quantitative assessment, an experienced medical professional also visually assessed the quality of the segmentations. We found that also in the visual analysis the performance of the networks was very high. However, while we saw excellent performance for large vessels, the performance in smaller vessels was less pronounced. While future networks should be improved regarding small vessel segmentation, the clinically most relevant vessels are the large vessels. Thus, we present evidence that already a relatively simple U-net architecture shows clinically highly relevant performance. Even higher performance can be expected using newer segmentation architectures, e.g., the MS-net (Shah et al., 2018). Also, when confronted with pathological cases, like cortical laminar necrosis and a rete mirabile, the performance of the network was limited. Here, there is a need for specifically tailored datasets being incorporated in the training samples. Taken together, the high quantitative and qualitative performance of the U-net are very promising for the development of new individualized precision medicine tools for stroke and cerebrovascular disease in the clinical setting. Vessel parameters could augment predictive models in cerebrovascular disease (Feng et al., 2018; Livne et al., 2018; Nielsen et al., 2018).

Our results confirm previous works in the field of vessel segmentation. Recently, pre-prints on ArXiv.org have explored deep neural nets architectures for brain vessel segmentation in healthy subjects (Chen et al., 2017; Tetteh et al., 2018). The reported performance measures were comparable to our results. This confirms the advantages of deep learning approaches for vessel segmentation tasks. A current limitation, however, is the lack of a standardized labeled vessel imaging dataset. For other segmentation tasks, labeled datasets have been published in the past, usually within public competitions (website: grand-challenges)^7^. A big advantage of such datasets and the competition framework is that it makes models comparable. If different datasets and especially different types of labeling are used, results can be roughly compared qualitatively, but a direct quantitative comparison cannot be performed. If, however, models cannot be compared, the translation of these new methodologies into clinically usable tools is strongly hampered. Thus, the medical machine learning community needs to address this issue by providing standardized datasets of vessels both with and without pathology for segmentation tasks in the established form of competitions to allow proper benchmarking of methods. The authors of this study would happily contribute to such an effort.

We chose a simple architecture that closely followed the suggested U-net by Ronneberger et al. This resulted in roughly 30 million parameters. Promisingly, we found that a U-net with half of the convolution channels—coined half U-net—showed comparable performance, and yet consisted of only roughly 8 million trainable parameters. Naturally, the training of the half U-net can be done much faster, in our case in 50% of the time. This might be attributable to a limited variability of brain vessels as captured by the dataset that allows less complex architectures to perform comparably. This is also shown by the fact that we used a simple 2D-patch approach with success. It seems that certain segmentation tasks do not necessarily need complex models and 3D approaches to reach sufficient performance. However, a systematic assessment of the necessary model complexity, particularly the number of feature channels, and 2.5D and 3D approaches is warranted in future studies to find the optimal approach for vessel segmentation. Especially for small vessels detection, such approaches might be promising. The optimal patch-size was identified for both architectures as the largest tested value of 96 × 96 voxels. This may imply that a larger patch-size may be more beneficial for the segmentation task. Such future optimization could be potentially done using advanced hardware or by increasing the patch-size on the expanse of the batch-size.

An important part of the model training is the augmentation of the data. CNNs—and the encoder part of the U-net utilizes convolutional layers—are not equivariant to certain transformations, especially not rotations. It is thus absolutely essential to perform augmentations, especially when (relatively) few training examples are available (Ronneberger et al., 2015). The main principle of augmentation is that the newly generated data represents new information that would occur in the same domain where the original images stem from. We chose in our work the *ImageDataGenerator* implemented in Keras. It is a multi-purpose augmentation tool, that on one hand will generate helpful new training examples with high likelihood, but on the other hand will be naturally less specific than individually tailored augmentation strategies. Here, a highly promising approach is the application of Generative Adversarial Networks (GANs) for data augmentation, e.g., by Antoniou et al. (2017). The adversarial generative and discriminative networks ensure—if mode collapse can be avoided—that a large variety of new data is generated which all lie in the same domain as the original data. Such images would allow ideal augmentation for any segmentation task.

Generalization of our findings to other vessel segmentation tasks signifies an important implication of our work. While it is possible to achieve high performance for vessel segmentation with hand-crafted features and parameters optimized for a special case, e.g., for CT-angiographies (Meijs et al., 2017), the development of such methods is time-consuming and a transfer of these results to images from other sources and other organs is hard to perform. In the case of a well-trained U-net, the convolutional layers have already learned the features necessary for the detection of vessels. Thus, it is possible to train new highly performant models for so far unseen vessel images by freezing the convolutional layers and by focusing the training on the rest of the model. This method is called “transfer learning” (Oquab et al., 2014) and requires only a few labeled datasets for each new source. Consequently, potential new tools can easily be adapted to various scanner settings, imaging modalities and even new organs, which is necessary for broad clinical adaptation and multicenter imaging studies.

We assessed model performance based on three different measures: First, the Dice coefficient. Mathematically it is equivalent to the F1 measure and thus the harmonic mean of precision and recall (Taha and Hanbury, 2015). It is a widely used measure for segmentation tasks and its popularity is explained by its insensitivity to background voxels, its easy interpretability and its customizability to improve learning in hard-to-segment regions (Shah et al., 2018). Together with patch extraction, the use of the Dice coefficient allowed us to alleviate the imbalanced sample distribution in our dataset, as only 0.9% of all voxels in the brain depict vessels. However, based on theoretical considerations, the Dice coefficient is limited when assessing the validity of vessel segmentations (Taha and Hanbury, 2015). For example, since vessels are narrow and elongated, segmentations errors can quickly lead to loss of overlap. However, once no overlap exists, the Dice coefficient cannot distinguish whether a segmented vessel is closer (better segmentation) or further away (worse segmentation) from the ground truth. Here, distance based measures are better suited (Taha and Hanbury, 2015), as they take into account the spatial position of voxels. Thus, we used two additional distance-based measures, the 95HD and the AVG. The plain Hausdorff distance was avoided due to its sensitivity to outliers. Promisingly, we saw a tendency that the models optimized by distance-based measures show improved results. It can thus be anticipated that customized loss functions incorporating distance-based measures will improve the performance of deep learning models for segmentation. Thus, future works should first systematically assess which metrics are best suited for brain vessel segmentation and then develop a customized loss function for vessel segmentation.

A special focus of our work was the selection of the dataset. First, we used patients with pathology, in our case cerebrovascular disease. The vessels of such patients are more challenging to segment owing to stenoses and occlusions, old infarcts and small vascular networks (“rete mirabile”). Thus, our results are more representative of the clinical challenges than the results of works using the data of healthy patients. And indeed, we found that special pathological cases like cortical laminar necrosis and a rete mirabile were challenging for the network and need to be focused in future works. In summary, our work serves as the starting point to develop new pathology-tailored models which are applicable in the clinical setting. In their training, random patch extraction should be avoided, and patch selection should be focused on the special cases identified in our study. Second, we labeled 66 patients, which is—in medical imaging—a large number of patients. This number is roughly double to triple of the number of the datasets used by Ronneberger et al. in their original U-net paper (Ronneberger et al., 2015). Since the U-net is tailored for use with limited data, our number of patients should allow for strong generalization and this is reflected by our high performance. Lastly, we invested a large effort into the labeling of the dataset. Every patient scan was labeled by a medical researcher and independently checked by 3 others medical researchers, 2 amongst them expert readers. It is very encouraging that two-digit numbers of high-quality labeled medical imaging data are sufficient to achieve very strong segmentation results with modern deep learning architectures. Labeling of such a number of patient scans is achievable in a justifiable time and opens the door for the development of high-performance models for the clinical setting for any medical segmentation task. It is to be expected that such models will soon be translated into applicable tools and will be available for research and the clinical setting.

Our study has several limitations. First, we used a monocentric dataset. Thus, imaging parameters and scanner parameters were fixed. In an intra-modal analysis, i.e., TOF-images from a different scanner with different parameters, the generalization performance was very good. In the inter-modal analysis, however, applying the models on MPRAGE-angiography images, the performance was considerably inferior. For the applicability in the clinical setting, two different strategies can be envisioned: (1) Since clinical on-site postprocessing is tied to the scanner-vendor and software, segmentation products tuned for vendor-specific sequences and parameter-ranges are possible and lack of generalization is unproblematic. (2) For development of a vendor-independent pipelines, clinical segmentation algorithms need to cover a much broader range of image variability. Here, a large number of varied datasets needs to be used for training of single models or model zoos in the future. Second, we reconstructed the images on the patch level and did not perform an algorithm-based optimization of the whole reconstructed vessel tree. Here, future works can explore for example recurrent neural networks, especially architectures with long short-term memory (LSTM) layers. Applying these techniques, an increase of small vessel segmentation performance might be possible. Third, also, the patch size was limited due to hardware constraints potentially reducing the performance of the network where more context is needed. Fourth, we performed an exploratory qualitative visual analysis by one medical expert. Future clinical assessments of different models should include a systematic quantitative rating by multiple medical expert readers, which exceeded the scope of the present work.

## Conclusion

In conclusion, a U-net deep learning framework yielded high performance for vessel segmentation in patients with cerebrovascular disease. Future works should focus on improved segmentation of small vessels and removal of artifacts resulting from specific cerebrovascular pathologies.

## Author Contributions

ML, TK, JS, JDK, KH, DF, and VM: concept and design; VM and JS: acquisition of data; ML, JR, AT, JDK, KH, and VM: model design; ML, JR, OA, EA, DF, and VM: data analysis; ML, JR, AT, TK, JS, JDK, KH, DF, and VM: data interpretation; ML, JR, OA, AT, EA, TK, JS, JDK, KH, DF, and VM: manuscript drafting and approval.

### Conflict of Interest Statement

While not related to this work, JS reports the following board memberships, consultancies, and/or payments for lectures including service on speaker's bureaus: Boehringer-Ingelheim, Sanofi, Bayer, Pfizer, and Maquet. The remaining authors declare that the research was conducted in the absence of any commercial or financial relationships that could be construed as a potential conflict of interest.

## Footnotes

^1^WHO EMRO Stroke, Cerebrovascular Accident | Health Topics. Available online at: http://www.emro.who.int/health-topics/stroke-cerebrovascular-accident/index.html (Accessed July 14, 2018).

^2^https://surfer.nmr.mgh.harvard.edu/fswiki/mri_nu_correct.mni (Accessed February 7, 2019).

^3^https://fsl.fmrib.ox.ac.uk/fsl/fslwiki/BET/UserGuide (Accessed July 14, 2018).

^4^Neuroimaging in Python-Pipelines and Interfaces-Nipy Pipeline and Interfaces Package. Available online at: https://nipype.readthedocs.io/en/latest/ (Accessed July 14, 2018).

^5^https://www.mevislab.de/ (Accessed July 14, 2018).

^6^http://www.itksnap.org/pmwiki/pmwiki.php (Accessed July 14, 2018).

^7^https://grand-challenge.org/All_Challenges/ (Accessed July 14, 2018).
